# Tuberculin and Immunity

**Published:** 1912-12

**Authors:** John Wm. Taylor


### TUBERCULIN AND IMMUNITY.

BY

JOHN WM. TAYLOR, M.D.

IN the new Insurance Act tuberculosis, especially pulmonary tubercle, is a marked feature, and as the study of tuberculin treatment is prominently before the profession at the present time, a brief consideration of some explanations of its action, as they strike a general practitioner, may prove of interest; but in this matter, and not only in questions of mere detail, medical men are by no means of one mind.

Let us first briefly consider some of the theories which underlie this application of vaccine therapy. It may be that there are still some practitioners who are not quite clear as to the difference between a "serum" and a "vaccine."[1]

*Serum Treatment.*—This form of treatment is most clearly illustrated and seen at its perfection in the case of diphtheria antitoxin, which is produced in the horse in response to injections of diphtheria toxin. It is accompanied by an increase of globulin content in the serum and diminution of the albumen content as the globulins rise.[2]

Later investigations have made it possible to concentrate the globulin content—containing the antitoxin—by precipitation, and to diminish the albumens in the antitoxic serum.[3]

On injecting the diphtheritic antitoxin the form of immunity produced is termed "passive immunity," as the tissues of the infected animal do not, themselves, form the antitoxin, which is introduced from without. It is maintained by some that the toxin, when neutralised by antitoxin, can stimulate the tissues of the infected animal to form antitoxin.

The antitoxin has no direct action upon the bacteria, although when the toxin they produce is neutralised, they tend to disappear.

The danger of serum disease (anaphylaxis) from repeated doses of serum, administered after a certain time, is the great drawback to all treatment by sera. This is due to the foreign albumen contained in the serum, and is not a part of its antitoxic character.

The effects of diphtheria antitoxin are well known, and its results in the treatment of diphtheria of the highest value, but unfortunately the value of antitoxin is mostly limited to diphtheria; with tuberculosis we are dealing with quite a different order of things. Diphtheria toxin is an extra-cellular or secretory poison; the poisons of tubercle, though possibly in part secretory, are now generally believed to be of the nature of endotoxins or intra-cellular material.[4]

*Vaccine Treatment.* — Whether bacillary bodies contain protein material apart from their endotoxins has not yet been definitely proved. In all bacterial bodies after removal of toxins and endotoxins a certain protein residue is said by some to remain. The nature of this residue has been called by Buchner " bacterial protein." The nature of the bacterial proteins is by no means clear, and it is doubtful whether the separation of these substances from the endotoxins can be upheld.[5] It has been found that extracts from various dead bacteria possess ferment properties in excess of those manifested by the same organisms when living.[6]

T.A. (Tuberculin Koch) is protein material obtained from pure cultures of tubercle bacilli, with extractions from the bacillary bodies. T.R. consists for the most part of extracts from triturated bacillary bodies. T.O. prepared from culture fluid represent a germ-free filtrate of T.B. cultures in bouillon, containing exo-toxins only. Bacillary emulsion (B.E.) represents a certain quantity of triturated T.B. in 8 per cent. saline solution, the bacilli distributed by shaking and centrifugalisation, the supernatant fluid forming a very fine emulsion. A dose of tuberculin injected into a normal person, that is to say a person never having suffered from tuberculosis, usually produces no reaction: some few individuals give a reaction who are not tuberculous,[7] and in advanced cases of tuberculosis no reaction occurs. But if injected into an individual who is suffering from tuberculosis, or has in the past suffered, what is known as " a reaction " occurs. This reaction may be focal and general, or focal only, or general only.

To what is the reaction due ? The generally accepted explanation is as follows : Tuberculin is a foreign albumen, and when a foreign albumen is introduced into the body tissues at the first no reaction occurs, but the formation of " lytic bodies " (hyper-sensitiveness) takes place.[8] These " lytic bodies " are ready to split up any further amounts of the specific foreign protein that may be introduced later ; by such action the foreign protein is made more toxic, and the reaction occurs. This explanation applies also to auto-inoculation phenomena.[9] Within favourable bounds the result of the reaction is limited to such an amount of increased tissue reaction around the focal lesions as may cause sufficient hyperæmia to carry increased amounts of antibodies in the serum and so favour healing; but in the production of the form of immunity aimed at in the treatment we are dealing with bacillary lysis—the breaking down of tubercle bacilli, and the liberation of endotoxins (tuberculin) in more or less unmeasureable amounts—the production of more tuberculin than very often is good for the patient, and added to any auto-inoculation that may already be in action.

In administering tuberculin, in whatever form, we are using a toxic compound, or what will become a toxic compound by lysis, in the hope that the reaction may be well within bounds, beyond which the possibilities of evil are limitless.

It appears then, from the first, that tuberculin treatment is not to be entered upon inadvisedly. What indications are there to mark the way? They are few and uncertain. The agglutination test is not entirely satisfactory. The fixation of complement test has often failed to be of any value. The opsonic test index is certainly the best, though laborious. The worst test of all is the production of reactions. Sahli is very emphatic in his statement that tuberculin dosage should be so regulated that as far as possible both focal and general reactions are avoided, and further, he says, "But I am absolutely opposed to diagnostic tuberculin injections." He decides that no definite conclusions can be drawn from either a negative or positive result, but apart from this he considers the risk sufficient argument for their rejection.[10]

Hyslop Thomson, in a recent work, suggests 0.1 cc. of either tuberculin diluted with a 30 per cent. solution of glycerine in water as an initial dose, and a rise of not less than 1° F. to be regarded as a diagnostic reaction. If no reaction, then after fourteen days 0.2 cc. If this fails further doses may be given at intervals. It is commonly considered that these amounts are entirely unjustifiable.

The important point to bear in mind when considering the use of tuberculin in fairly large doses for diagnostic purposes is (1) the large percentage of persons in these latitudes who at one time or another have been tubercular. Nageli [12] and Burckhardt [13] have proved the presence of small tubercular foci in nearly every adult body, healed in ways which were not clinically perceptible. As it is almost impossible ever to say during life whether a lesion is absolutely cured or not—the finding of T.B. in sputum not always obtainable—the test of a large dose of tuberculin may, in many cases, light up a quiescent lesion and result in disaster.

Many cases discovered by skilful diagnostic methods may be doing very well, and to interfere by inoculations may be meddlesome and dangerous.

Several forms of *serum treatment* for tuberculosis have been put forward by various workers. It is probable that antitoxin is concerned in the cure of tuberculosis, but antitoxin has its chief use in diseased conditions caused by exotoxins; the general opinion now is that endotoxins are the poisons chiefly concerned in tubercle, and antitoxins for such toxins are formed in the tissues locally, and are not present in any large quantities in the serum, and so phylogenetically adapted to local defence. The question of histogenous immunity may have some bearing upon the question. It appears that the path of active immunity, though "a thorny" one, is the correct one to follow.

Spengler [14] produces what he calls a solution of the tuberculosis immune substances (Immune Korper) which he calls I.K., by inoculation, intra-muscular, of virulent cultures in rabbits. He also adds "pus exciters and other bacteria found in association with tubercle bacilli." [15]

After testing, the blood is collected in a vessel containing an acid-carbol-sodium-chloride solution. He considers the presence of lactic acid prevents anaphylaxis. He claims that the serum contains both lysins and antitoxins, that I.K. produces passive immunity, and, as a secondary action, active immunity; the active immunising agents being formed in the patient's body by the action of the antitoxins upon the toxins, the toxophore groups being neutralised, these neutralised toxins possessing the power of stimulating the production of immune substances. Opinions are divided as to the efficacy of this form of treatment. Many authorities consider I.K. to be inert. Roepke concludes that the results obtained with I.K. treatment at Davos are due to Alpine climate only.

*Some practical considerations.*

Tuberculin used in apyretic and early cases is of value. Very careful recording of temperature per rectum is indispensable. All reactions should be studiously avoided—very small doses used in the first instance.

A mixed infection is a contra-indication.

The diagnostic use of tuberculin as an injection is dangerous.

The tuberculins of most value are those containing extracts of bacillary bodies. Sahli highly recommends tuberculin Beraneck, T.Bk.

Tuberculin possesses no direct healing power, and its application is different in principle from that of a true specific.

The presence of T.B. in the sputum is the only true and pathognomonic sign of pulmonary tuberculosis in the living.

A very large number of individuals possess cured tubercular lesions which have never caused recognisable clinical symptoms. Up to the present the only method of treatment attainable is that of active immunity, the all-important feature being the capability of the body tissues to react (power of resistance).

It appears that in many cases the use of tuberculin is unwarrantably in advance of the present state of knowledge of the problems of immunity.

### REFERENCES.

[1] J. Freeman and J. Ritchie, *Brit. M. J.*, 1912, ii. 1293, 1298.
[2] Hiss and Atkinson, *J. Exper. Med.*, 1900, v. 47.
[3] Hiss and Zinsser, *Text-book of Bacteriology*, 1910.
[4] *Ibid.*
[5] *Ibid.*
[6] J. W. Taylor and I. Walker Hall, *J. Path. and Bacteriol.*, 1912, xvii. 121.

[7] Sahli, *Tuberculin Treatment*, 1912.
[8] Wolff-Eisner, *Clinical Immunity*, 1911.
[9] *Ibid.*
[10] Sahli, *Tuberculin Treatment*, 1912.
[11] Hyslop Thomson, *Treatment of Consumption*, 1912.
[12] Nageli, "Ueber Haüfigkeit der Tuberculose," *Virchow's Arch.*, 1900, clx. 426.
[13] Burckhardt, *Zeit. f. Hyg.*, 1906.
[14] Spengler, *Tuberkulose u. Syph. Arb.*, 1911.
[15] Fearis, *I. K. Therapy*, 1912.

